# A Review of the Chemistry and Biological Activities of *Acmella oleracea* (“jambù”, Asteraceae), with a View to the Development of Bioinsecticides and Acaricides

**DOI:** 10.3390/plants11202721

**Published:** 2022-10-14

**Authors:** Eleonora Spinozzi, Marta Ferrati, Cecilia Baldassarri, Loredana Cappellacci, Margherita Marmugi, Alice Caselli, Giovanni Benelli, Filippo Maggi, Riccardo Petrelli

**Affiliations:** 1 School of Pharmacy, Chemistry Interdisciplinary Project (ChIP), University of Camerino, Via Madonna delle Carceri, 62032 Camerino, Italy; 2 Department of Agriculture, Food and Environment, University of Pisa, Via del Borghetto 80, 856124 Pisa, Italy; 3 Centre of Plant Sciences, Scuola Superiore Sant’Anna, Piazza Martiri della Libertà 33, 56127 Pisa, Italy

**Keywords:** spilanthol, biopesticide, eco-friendly insecticide, acaricidal activity, moth, mosquito, stored-product pest, ticks, non-target toxicity

## Abstract

Human pathologies, environmental pollution, and resistance phenomena caused by the intensive use of chemical pesticides have shifted the attention of the agrochemical industries towards eco-friendly insecticides and acaricides. *Acmella oleracea* (L.) R. K. Jansen (jambù) is a plant native to South America, widely distributed and cultivated in many countries due to its numerous pharmacological properties. This review analyzes literature about the plant, its uses, and current knowledge regarding insecticidal and acaricidal activity. *Acmella oleracea* has proven to be a potential pesticide candidate against several key arthropod pest and vector species. This property is inherent to its essential oil and plant extract, which contain spilanthol, the main representative of *N*-alkylamides. As a result, there is a scientific basis for the industrial exploitation of jambù in the preparation of green insecticides. However, studies related to its toxicity towards non-target species and those aimed at formulating and developing marketable products are lacking.

## 1. Introduction

The use of pesticides has strongly expanded in recent years, inducing side effects on human health. Insecticides and acaricides of synthetic origin can be toxic to useful arthropods and adversely affect the environment. Moreover, they can lead to resistance phenomena in arthropod target populations as they are usually made up of a single active ingredient. To face the development of insecticide and acaricide resistance, it is necessary to focus on eco-friendly pest and vector management strategies [1,2,3,4], which are aimed at reducing the exposure of non-target species to hazardous chemicals [5,6,7]. Moreover, in recent years, costumers are shifting their attention towards food safety, resulting in an increase in the demand for biological food and organic products. Agrochemical industries are responding to the current needs of the global market, expanding the research and development of eco-friendly pesticides [8]. Essential oils (EOs), plant extracts, and botanical compounds have been highlighted as valuable candidates for bioinsecticide development that are up to sustainable biological standard. Currently, biopesticides cover only 5% of crop protection products, but they are increasing by about 10% per year. Unfortunately, nowadays, they are only used as an aid to traditional products. The main goal is to fully substitute synthetic substances with natural ones that are safer and economically convenient [9]. Agrochemical industries are working on active ingredients that are effective against more target species and can be easily obtained. This should allow the use of the lowest amount of the product and, accordingly, lead to greater economic and ecologic advantages [10].

*Acmella oleracea* (L.) R. K Jansen is the most common cultured species of the *Acmella* genus and belongs to the Asteraceae (Composite) family. It is a flowering herb native to South America, where is called jambù, and nowadays, is used all over the world for food, cosmetics, pharmaceutics, and pest-management purposes [11,12]. This plant contains a wide array of secondary metabolites, the *N*-alkylamides being mainly responsible for its biological properties. Spilanthol, which is included in this class of compounds, has shown, among others, a high pesticidal efficacy [13] due to its good ability to penetrate in the insects affecting the central nervous system (CNS) [12]. Agrochemical industries and research groups have also further investigated the insecticidal and acaricidal potential of *A. oleracea* extracts and EO against several target species of agricultural (e.g., moths, aphids) [14,15] and medical importance (e.g., ticks, mosquitoes, houseflies) [14,16,17,18,19]. Moving plant-based insecticides from the laboratory to the field is still difficult due to regulatory issues, though the non-target impact should be deeply studied. Although *A. oleracea* products have attracted significant attention in recent years, to our knowledge, there is no review available on their insecticidal and acaricidal efficacy. On the above, this work can be useful to identify the knowledge gaps of the insecticidal and acaricidal potential of *A. oleracea* and make it more readily available to scientists around the world who are working in the areas of bio-pesticide development.

In the following paragraphs, we reviewed international literature about the insecticidal and acaricidal activity of *A. oleracea* extracts, EO, and its main constituents. Side effects on non-target species as well as challenges for real-world employ have also been analyzed.

## 2. Systematics

There is confusion in the literature regarding the name of the genus and species of *A. oleracea*, and the genus *Acmella* is often taxonomically mistaken with the genus *Spilanthes.* This confusion leads to several overlapping botanical names, resulting in numerous taxonomic revisions and reclassifications [20]. In fact, this plant is sometimes reported as *A. oleracea* (L.) R. K Jansen [21], *S. oleracea* L. [22], or *S. acmella* (L.) L. [23].

However, different morphological, chromosomal, and phytochemical studies indicate that the genera *Acmella* and *Spilanthes* are quite distinct, thanks to the presence of eight different morphological features and characteristic chromosomes [20]. The actual accepted name of jambù is *Acmella oleracea* (L.) R. K. Jansen, belonging to the genus *Acmella*, which is included in the Asteraceae family [24].

## 3. Habitat and Distribution

It is suggested that the origin of *A. oleracea* is linked to the cultivation of *Acmella alba* (L’Hér.) R. K. Jansen in central Perù [25]. However, in some cases, its nativity has been attributed to Brazil, where the plant is called jambù and is grown as an ornamental or medicinal plant [26,27]. Even though its origin is unclear, it is certain that it is cultivated throughout the year, and is widespread as a crop in different parts of the world. Cultivation of *A. oleracea* was introduced through the tropics and then reached the subtropics [28]. As a result, the plant is found in the USA; South America, particularly in Mexico, Perù, Brazil, and Bolivia; northern Australia; Africa; and Southeast Asia: Bangladesh, Nepal, India, and Sri Lanka, Indonesia, and China [13,14,25,29,30]. *A. oleracea* is a perennial herb in the Mediterranean area [12]; its spread is due to its easy cultivation and easy growth in the wild [31]. In fact, thanks to wide cultivation, it has been naturalized in tropical east Africa, as it can be found in lake margins or equally humid areas and secondary woodland margins in Uganda, Kenya, and Tanzania [28]. Its distribution can be explained by the tendency of jambù to prefer full sun exposure, being sensitive to frost and perennial in warmer climates [32]. The plant is mainly found at low altitudes, up to 1200 m a.s.l. [28], and grows on drained and humus-rich soils [14]. It prefers humid environments and can be found in villages, pastures, rice paddies, and cultivated areas, along ditches, marshy meadows, open expanses, old glades, on open hillsides and rocky riverbanks, and along roadsides [14,32].

## 4. Morphological and Anatomical Features

**Figure 1:**
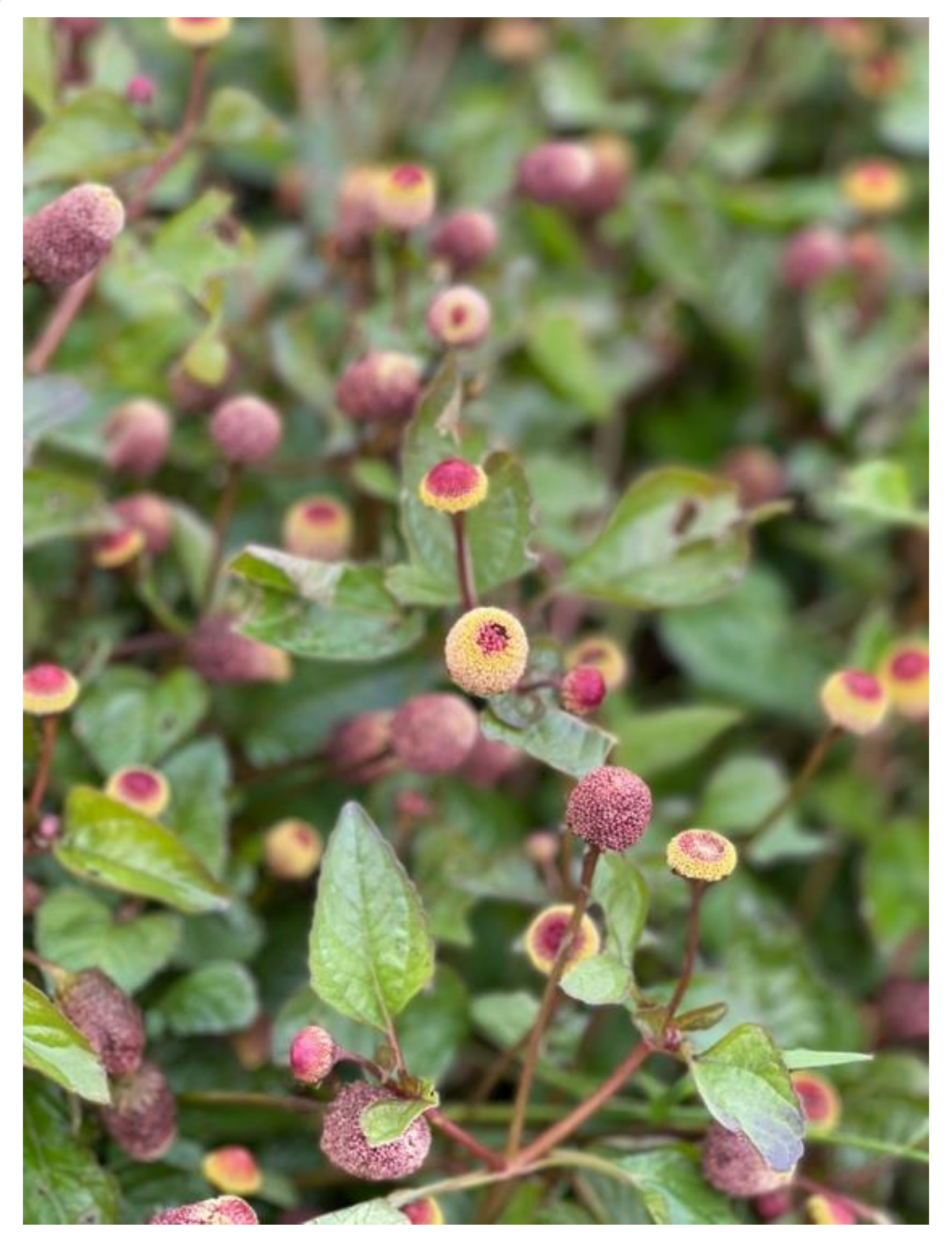
Heads and receptacles of *A. oleracea*.

*A. oleracea* is an annual plant [33] that grows up to 40–60 cm, usually showing decumbent to ascending stems, with no roots at nodes, showing a glabrous texture from green to red, and with a diameter from 2.5 to 7.5 mm. Its petioles can reach a length from 2 to 6.5 cm and are usually winged, glabrous, or lightly pilose. Leaf blades are ovate to deltate, and the capitula have a large and discoid shape, with a height from 10.5 to 23.5 mm and a diameter from 11 to 17 mm. The peduncles have a length and a diameter of 3.5–12.5 cm and 11–17 mm, respectively, and they are usually glabrous. The heads are discoid, and the receptacle, occasionally constituted by two heads, has a height of 8.3–21.5 mm and a diameter of 3.5–8.5 mm (Figure 1). *A. oleracea* yellow corollas have a length of 2.7–3.3 mm; the tube is usually 0.5–0.7 mm long and has a diameter of 0.2–0.4 mm. The plant has 1.4–1.7 mm-long stamens and black anthers. The ciliate achenes have a length of 2–2.5 mm and a width of 0.9–1.1 mm, and the pappus is constituted by 2 bristles, one of 0.5–1.5 mm and the other of 0.3–1.3 mm [25].

## 5. Cultivation and Micropropagation

**Figure 2:**
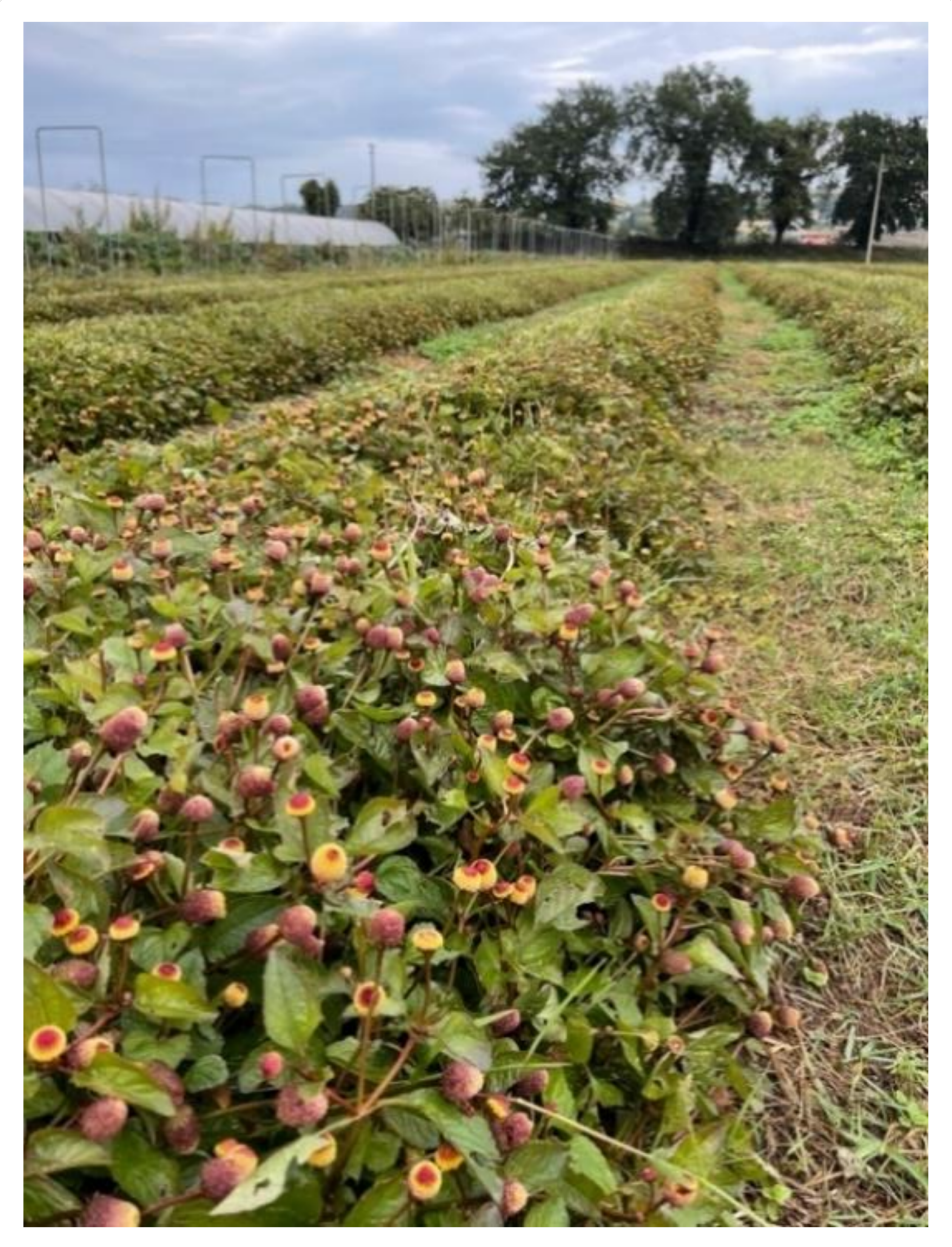
*A. oleracea* field cultivation.

*A. oleracea* is typically cultivated in the field by direct sowing. It prefers moist and clayey soils, rich in organic substances, among which nitrogen plays a fundamental role [34]. Its crops need some treatments; they must be constantly watered, to prevent them from drying out quickly, and they must be exposed to the sun as they cannot grow in full shade. Although the optimal temperature for seed germination is 21 °C, this plant can also be cultivated in winter if protected by glass. It is preferable to sow it in April, while flowering begins in July and ends in October. The phenological cycle of the plant is around 6/8 months. It propagates better by seed or cutting; it does not frequently root at the nodes; it does not spread far, and, for this reason, dense crops are recommended (Figure 2) [12,29,35].

*A. oleracea* shows several functional properties, and, as a result, the interest of cosmetic, pharmaceutical, and agrochemical companies has increased over the years [36]. Consequently, various studies have been carried out, both to increase the productivity of the crop and to standardize the quality and quantity of its main secondary metabolites. A recent study [37] evaluated the influence of bovine manure and urea on the plant yield, which was greater with the use of mineral fertilizer, compared to the manure one. The influence of a mixture of leaves of *Syzygium malaccense* (L.) Merr. & L. M. Perry, *Inga edulis* Mart., *Mangifera indica* L., and *Zoysia japonica* Steud. and vermicompost was evaluated, and the best agronomic yield of the jambù was obtained with the application of 10 kg m^−2^ of the organic fertilizer, with greater productivity in the cultivation carried out between May and June [38]. Regarding the influence on metabolite production, Borges et al. observed an increase of 31.6% in spilanthol content and 16.8% of flavonoids in the inflorescences and higher levels of total phenols, carotenoids, spermidine, and spermine in the leaves and flowers of jambù after a treatment with an organic fertilizer compared to conventional fertilizer use [39,40].

The action of biostimulants has also been tested to increase the production of alkylamides. Crop growth increased after the use of both a triacontanol-based blend and a plant tissue extract, but the total content of *N*-alkylamides and polyphenols was not affected [41].

Recently, methods other than conventional crops have also been proposed. Although fertilizers were very useful for the increase of the *A. oleracea* productivity, some authors introduced hydroponic cultures to cope with crop losses caused by diseases and insects.

Several parameters of jambù hydroponic culture have been evaluated in response to an increasing concentration of nitrogen (N) in the nutrient solution, given its importance for optimal growth [37]. The change in N concentration resulted in an effect on growth, yield, and post-harvest quality and the optimal concentration for best performance was 21 mmol L^−1^ [42]. Furthermore, Nascimento et al. evaluated the influence of the type of cultivation, hydroponic or conventional, on the chemical profile of leaves, flowers, and stems of *A. oleracea*. The aerial parts showed a higher content of polyphenols and flavonoids in conventional crops than in hydroponic ones, except for flowers [34]. 

To find an alternative approach to the already described cultivation techniques of *A. oleracea*, many researchers established the optimal conditions for the micropropagation of this plant. It was firstly micropropagated using axillary buds as explants, on a Murashige and Skoog (MS) medium supplemented with 2.0 mg L^−1^ of *N*^6^-benzyladenine [43]. However, Malosso et al. utilized nodal segments in an MS medium supplemented with 0.1 mg L^−1^ kinetin [44]. An efficient and improved in vitro propagation method, using a transverse thin-cell layer (tTCL) culture system was also developed by Singh et al. In this case, nodal segments were used as explant source, and a MS medium with 5.0 mg dm^−3^ of 6-benzylaminopurine (BAP) was optimal for shoot regeneration [45]. Singh and Chaturvedi demonstrated for the first time the high producibility of a micropropagation method starting from cultures of nodal segments. MS medium supplemented with 5 μM BAP was best for recurrent shoot multiplication at a rate of 10 times every 5 weeks, and this shoot multiplication rate was maintained for nearly 1 year [46]. Moreover, Leng et al. carried out a study to detect the presence of spilanthol and other useful compounds from the mother plant, in vitro seedlings, callus cultures, and cultures in cell suspension. Through this study, spilanthol and *N*-isobutyl-(2*E*,4*Z*,8*Z*,10*E*)-dodecatetraenamide were firstly found in micropropagated plantlets [47]. A few years later, Franca et al. evaluated spilanthol content in *A. oleracea* plants obtained from three different cultivation conditions: in vitro, acclimatized and in-field and compared two different extraction techniques, such as maceration and microwave-assisted extraction (MAE). Results showed that the acclimatized plants were the richest in spilanthol, followed by the in vitro culture and, finally, the field material [48]. The aim of another study was to determine the best concentration of growth regulators for the in vitro cultivation of jambù and to elaborate an efficient micropropagation protocol. The best treatment was obtained in an MS medium with the use of 0.125 mg L^−1^ of the BAP, with an average of 2.2 sprouts/explant, an average height of 2.4 cm, and vigorous sprouts [49]. Table 1 summarizes the studies reported for *A. oleracea* cultivation.

## 6. Ethnobotanical Uses

The pharmacological uses with experimental support of *A. oleracea* are well known so far and have already been reviewed [50]. As a matter of fact, this plant has also been used for purposes other than medical ones. In ancient times, as early as 1700, it was cultivated as an ornamental plant in a botanical garden in St. Vincent, Kingstown [29], thanks to its attractive, large, and cylindrical heads characterized by red to purplish paleae, which then contrast with flowers ranging from yellow to cream [25]. In addition, the use of *A. oleracea* in the culinary field has been handed down from the most ancient tribal communities to today’s generations over the years, and its parts, such as the leaves and the tips of young shoots, are eaten both raw and cooked [13,28]. The first report on the taste of *A. oleracea* was made by Lamarck in his Encyclopedie Methodique [51], who noticed that the plant had a very spicy but unpleasant taste. Despite this, the plant is served in first and second dishes, especially in Brazil but also in Japan, India, and Indonesia, and added in salads thanks to its spicy aroma in the USA [52,53,54,55]. In Taiwan, jambù is traditionally cooked with duck, but over time, its use has been extended to rice dishes (Pará rice), pizza, and salads [53]. Moreover, it is used as a spice in some countries [56], and the extracts of the plant are used as food flavorings in kitchens all over the world [34]. The most widely reported use of *A. oleracea* is medicinal, especially against toothache [29], being described as a toothache plant [57,58,59]: it is applied directly to the pain site due to its anesthetic property [13] but also formulated in a tincture or in the form of an oral gel [50]. Usually, it is recommended to chew the roots, the leaves, and the flower heads, even if the capitula, especially if fresh, have a more marked effect [34]. Moreover, the decoctions of leaves and flowers are remedies for constipation, toothache, or stomatitis [35]. Besides being used as a remedy for mouth and throat disorders, *A. oleracea* is also used in the form of tea, while its extract is added to antiseptic products such as toothpaste and mouthwash [60]. The plant is also known to be used as an appetite stimulant in Brazil and Japan [46,61]. In Cameroon, it is used to treat snakebites, and in India, the flower heads are used to treat stuttering in children, while the leaves and flowers of the plant are used to treat leukorrhea in Bangladesh [13]. In India, jambù is used as an aphrodisiac to increase desire and improve a man’s sexual function [31]. Its range of applications in traditional medicine is wide and includes other uses such as tonic, anticonvulsant, antifungal, antiprotozoal, antidiarrheal, antimalarial, antiulcer, antipyretic, diuretic, anti-inflammatory, diuretic, and insecticide [27,31,62].

## 7. Secondary Metabolites

*A. oleracea* is a rich source of secondary metabolites, and its phytochemistry has been widely investigated. The work of Abeysiri et al. [63] revealed that alkaloids, flavonoids, saponins, steroid glycosides, and tannins are distributed in all parts of the plant. This study also revealed the major presence of phenolics in leaves and flowers rather than in stems. Going more in detail in the phytochemical composition of jambù, several triterpenoids have been found, such as 3-acetylaleuritolic acid, *β*-sitostenone, and stigmasterol. Moreover, steroidal glycosides, namely stigmasteryl-3-*O*-*β*-D-glucopyranoside and *β*-sitosteryl-3-*O*-*β*-D-glucopyranoside were identified. Furthermore, several phenolics have been detected such as vanillic, *trans*-ferulic, and *trans*-isoferulic acids [64]; scopoletin [64]; and fatty acids such as *n*-hexadecanoic and *n*-tetradecanoic acids [46].

**Figure 3:**
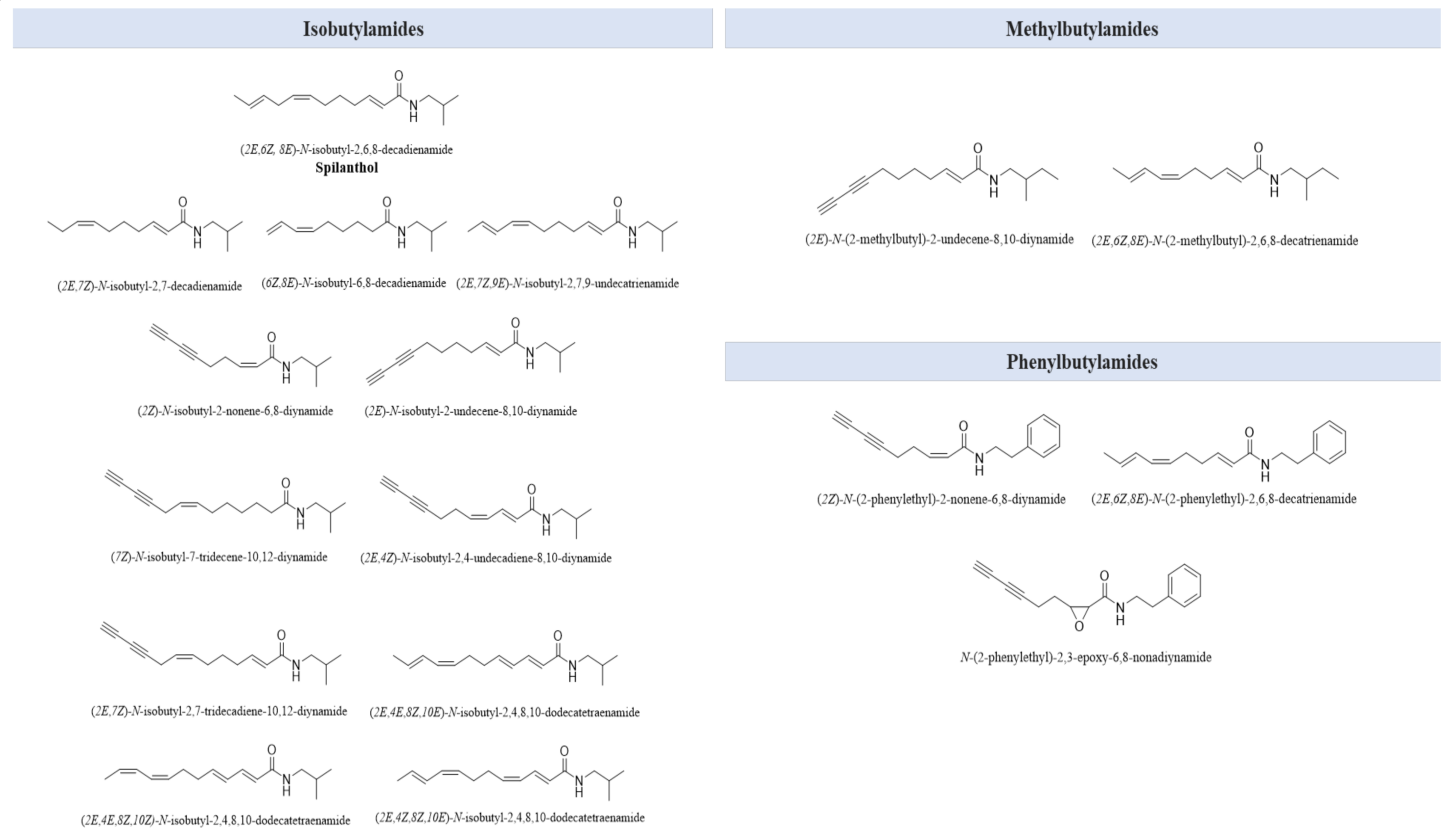
Main *N*-alkylamides found in *A. oleracea*.

*A. oleracea* also contains a volatile fraction, represented by the essential oil (EO) that can be obtained from its inflorescences and that is mainly characterized by *β*-pinene, myrcene, (*E*)-caryophyllene, caryophyllene oxide, germacrene D, *β*-phellandrene, spilanthol, and acmellonate [12,14,65]. Despite the richness and variety of secondary metabolites (Table 2), the compounds mainly responsible for its bioactivity are alkylamides, which are described as pseudo-alkaloids because of their structural similarity to this class of compounds. They are fatty acid amides, derived from a condensation reaction between a fatty acid chain and a decarboxylated amino acid. In fact, it is reported that there are several chemically distinct amine fractions that match to different fatty acids conducting to diverse and numerous *N*-alkylamides in *A. oleracea* [66]. However, the most abundant compound of this class is spilanthol, which is an olefinic *N*-alkylamide endowed with an isobutyl side chain. Spilanthol plays a role of primary importance, as it is responsible for the main biological activities and sensory effects characterizing jambù [27]. The *N*-alkylamides identified in *A. oleracea* are reported in Figure 3, and they are classified as isobutylamides, methylbutylamides, and phenylbutylamides according to the side chain bonded to the nitrogen atom of the amidic moiety [67,68].

## 8. Synthesis of Spilanthol

**Scheme 1:**
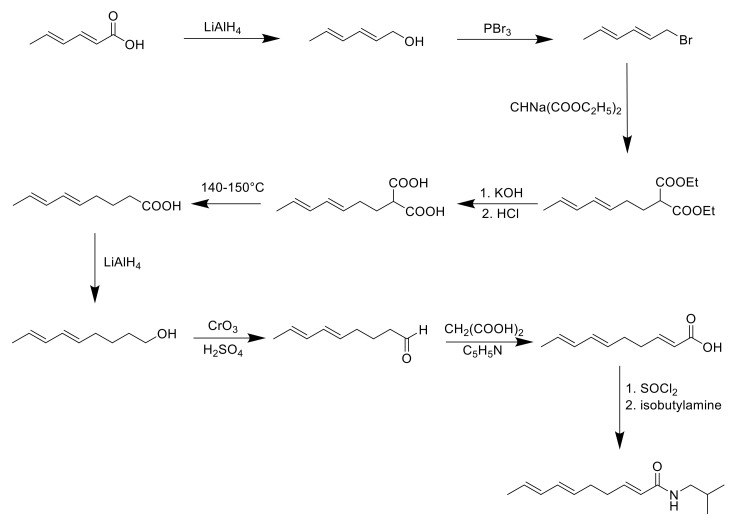
Synthesis of all-*E* spilanthol isomer [70].

As spilanthol is the key compound of *A. oleracea*, exerting most of the plant’s biological properties and being employable on an industrial level, the synthetic approaches have focused mostly on this metabolite. The first isolation of spilanthol dates to 1903 when Gerber isolated it from *S. oleracea* [69]. The isolation from a natural matrix, even after further optimization, was harsh and resulted in very low yields. For these reasons, the research focused on a synthetic alternative to obtain the pure compound. Already in 1955, Jacobson provided a nine-step synthesis for what spilanthol was thought to be; at that time, the stereochemistry of the natural compound was still unknown, and the process optimized by Jacobson led to the all-*E* isoform of spilanthol [70] (Scheme 1).

**Figure 4:**
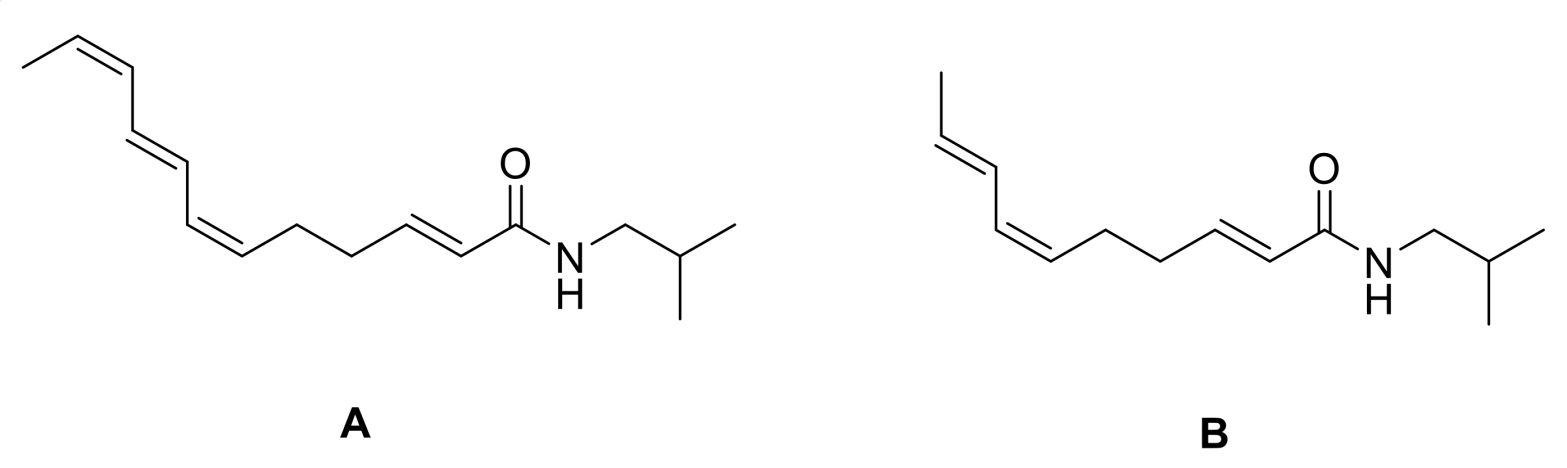
Neoherculin (**A**) and spilanthol (**B**) structures.

Only in 1963, Crombie et al. defined the exact stereochemistry of spilanthol. They confirmed the *E* conformation of 2-linkage through infrared evidence, and the analogy with neoherculin (Figure 4), whose stereochemistry was known, suggested an internal (6*Z*,8*E*) linkage. The (2*E*,6*Z*,8*E*)-trienamide stereochemistry was confirmed to be the exact one by a comparison of the melting point, infrared spectra, and biological activity with the isolated natural product, which had identical results [71]. 

**Scheme 2:**
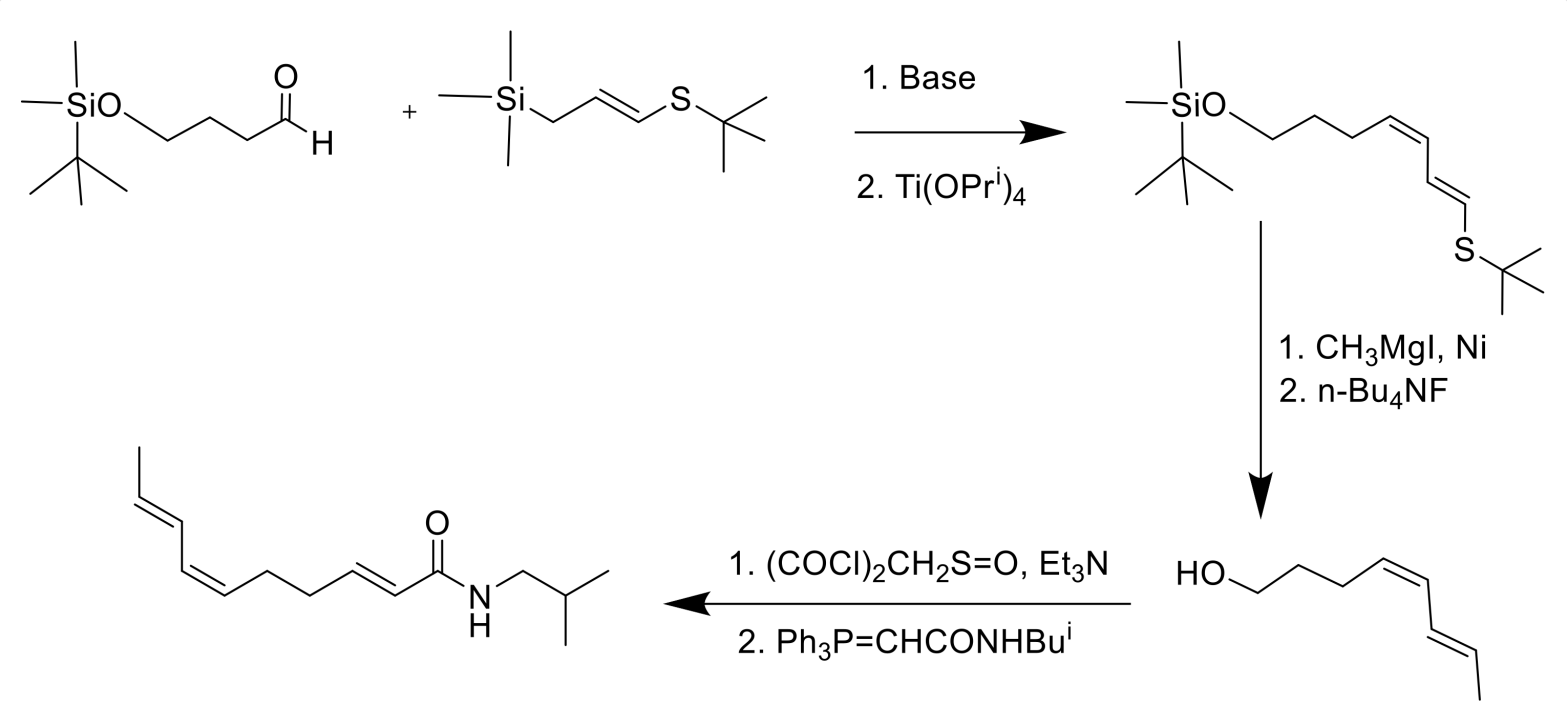
Ikeda et al. procedure using allyltitanium species as a catalyst [72].

**Scheme 3:**
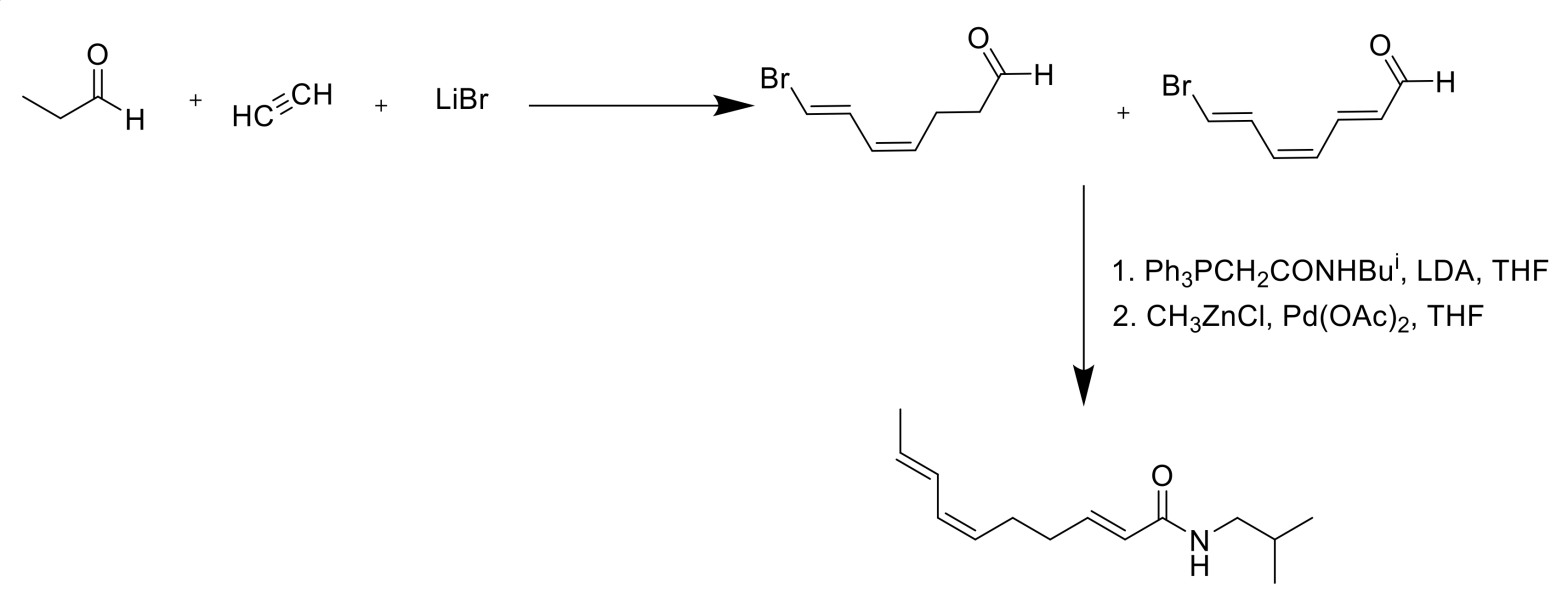
The shortest spilanthol synthesis reported in the literature by Wang et al. [73].

**Scheme 4:**
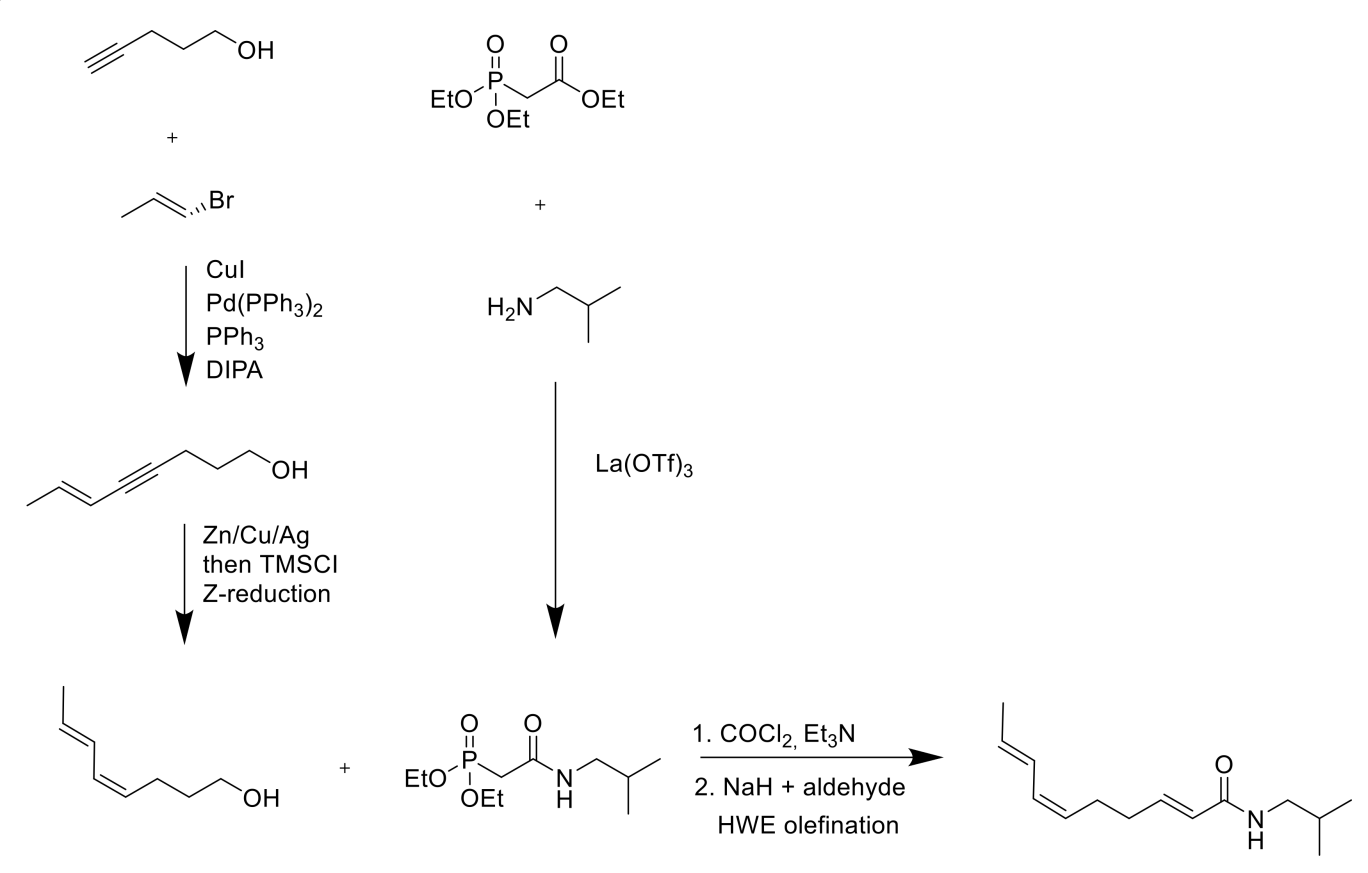
Synthesis route through HWE olefination [79].

Ikeda et al., years later, approached the synthesis using a convergent strategy adding an allyltitanium species over an aldehyde as a key step [72] (Scheme 2). The product was obtained in 88% purity. The shortest synthesis was ideated by Wang and co-workers in 1998 [73]: there, the crucial transformation employed was a cotrimerization of acrolein with two equivalents of acetylene, which assembled the conjugated diene fragment with the required geometry with 43% yield (Scheme 3). Unfortunately, acetylene is extremely flammable, and, on a large scale, the risk of explosion is likely to constitute an issue. In the first years of the 2000s, a series of patents from Takasago International Corporation [74,75,76,77,78] described alternative syntheses for spilanthol, characterized by simple purification processes but poor control over the exact double-bond formation; 78% of the final mixture constituted the desired product; 18% was the all-*E* isomer, and the remaining 4% was the (2*E*,6*Z*,8*Z*)-isomer. In 2018, Alonso et al. studied a 5-step synthesis using a Horner–Wadsworth–Emmons (HWE) olefination reaction for the 2*E* double-bond formation: this reaction allowed an easier purification of the product compared to the Ikeda and Wang procedures wherein the triphenylphosphine oxide produced by the Wittig reaction generated difficulties in the purification process [79] (Scheme 4).

**Scheme 5:**
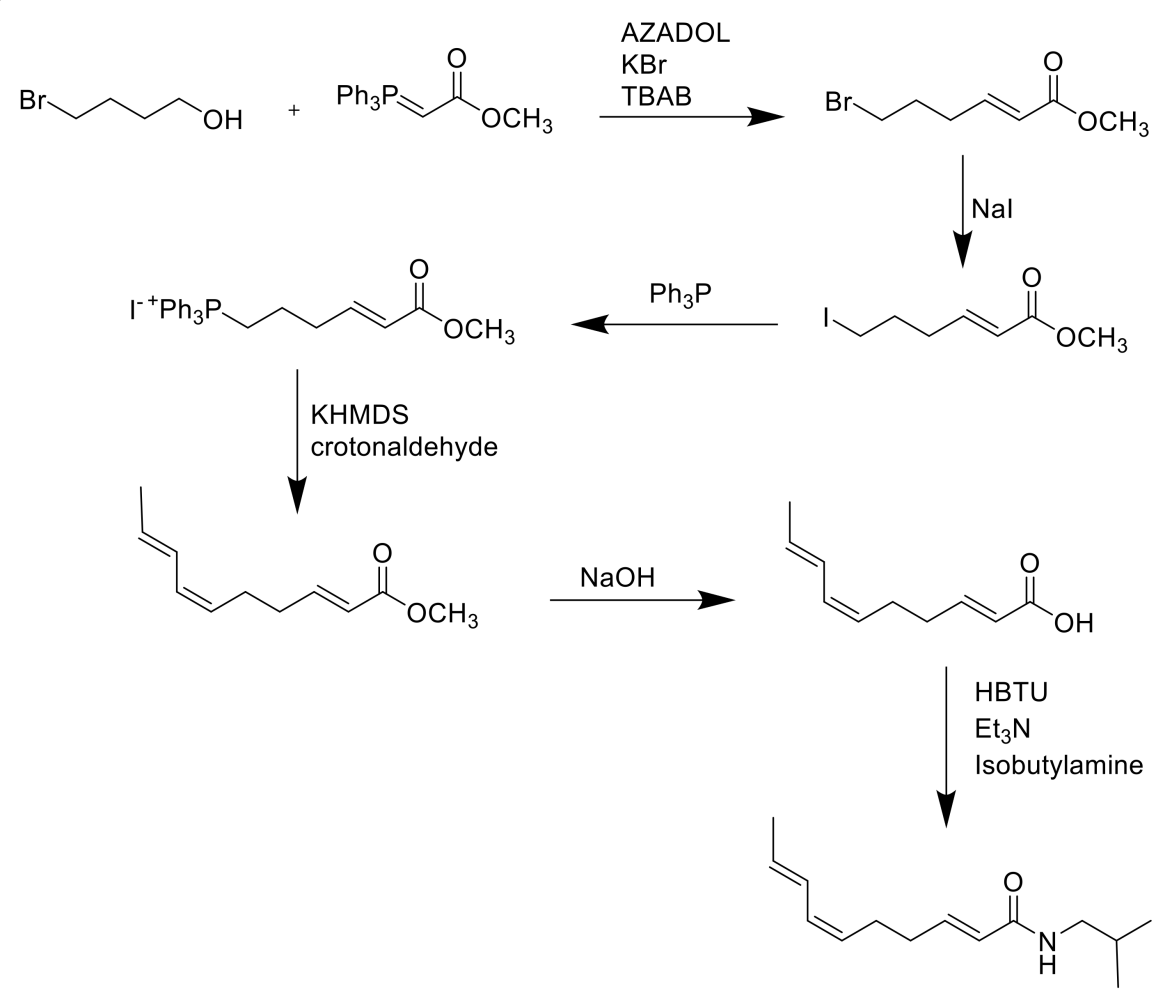
Synthesis of spilanthol through the employment of AZADOL as a catalyst of the stereoselective Wittig reaction [80].

The most recent attempt reported in the literature for the synthesis of spilanthol was performed by Nakamura in 2020, wherein a highly selective Wittig reaction produced the desired *Z*-form tetraene through the employment of newly synthesized phosphonium salt with low deliquescence and long-term stability. The final yield was 47% [80] (Scheme 5).

## 9. Modes of Action of Spilanthol

Spilanthol has several biological activities, carried out through different mechanisms of action. In this respect, its lipophilicity surely plays a crucial role for the properties demonstrated by this *N*-alkylamide. In fact, an in vitro permeability test showed that it can cross the CaCo-2 (immortalized cell line of human colorectal adenocarcinoma) cell monolayer cultures through passive diffusion. On the other hand, in vivo assays demonstrated its capacity to permeate the human skin and oral mucosa [81] and to reach blood circulation together with the crossing of the blood–brain barrier in studies on rats and mice [82]. Among the effects caused by spilanthol (also named affinin), the antinociceptive one is surely predominant. Déciga-Campos et al. [83] proved that this effect is linked to the activation of serotoninergic, opioidergic, and GABAergic systems and involves the NO/cGMP/potassium channel pathway. The anti-inflammatory action is also worthy of mention, and, in this case, spilanthol causes NO-suppressive effects but also protects from NO-dependent cell death. Moreover, it was demonstrated that this compound reduces the expression of iNOS mRNA and protein, which results in an anti-inflammatory effect [84]. Spilanthol also reduces the expression of pro-inflammatory cytokines PGE2, COX-2, and TNF-α, as well as MCP-1 and ICAM-1 expression, by inhibiting the NF-κB and MAPK signaling pathways and suppressing the monocyte adhesion to IL-1β-stimulated A549 cells [85]. The antifungal and antiaflatoxigenic properties of this *N*-alkylamide were demonstrated against *Aspergillus parasiticus* through the downregulation of the aflD and aflR genes, which are involved in the aflatoxin biosynthetic pathway, but also in the molecular mechanisms underlying the growth inhibition of *A. parasiticus* [86]. Spilanthol was also demonstrated to be a diuretic agent by the reduction of the basal phosphorylation level of NKCC2 in mouse kidney slices and in NKCC2-expressing HEK293 cells. This compound also has antimutagenic activity, which was demonstrated by its capacity to reduce 2AA- and NOR-induced mutations in strains of *Salmonella typhimurium* but also the mutations caused by 2-aminoanthracene (2AA) and the DNA damage linked to norfloxacin [87]. Spilanthol also displayed great insecticidal potential against different insect pests and vectors, and it can be considered as a promising active substance for manufacturing botanical insecticides. Electrophysiological studies indicated immediate hyperexcitation, followed by the complete inhibition of cockroach cercal nerve activity in insects treated with *A. oleracea* extract: this is evident from the abnormal movements, such as uncoordinated muscular activity [88]. This compound has good ability to penetrate insects, and, even if the mechanism of action has not been determined yet, spilanthol seems to affect the central nervous system because of some abnormal movements (i.e., not coordinated muscular activity) observed in the tested insects. In addition, the rapid mortality of larvae after a short exposure to spilanthol and other alkylamides demonstrated that they disrupt the processes of the histolysis of larval tissues. The hypothesis is that this compound also affects histolysis and histogenesis processes [12]. Similar amides from the Piperaceae showed promising insecticidal activity. In detail, piperamides from *Piper nigrum* L. demonstrated insecticidal properties against different mosquito larvae. These compounds displayed various mechanisms of action, such as the neutralization of insects detoxifying the system interfering with the activity of oxidoreductase, monooxygenase, and glutathione S-transferase. Moreover, they exhibited neurotoxicity through the activation of the nicotinic acetylcholine receptors and the disruption of embryonic development and antifeedant activity [89]. Given the structural similarity with piperamides found in black pepper extracts, spilanthol’s mode of action could involve similar pathways, and more studies should be performed to support this hypothesis.

## 10. Biological Activities

*A. oleracea* has shown a wide range of applications due to its multiple biological activities. The main pharmacological actions studied up to 2013 have already been summarized [13]. Over the years, many authors have evaluated already known properties on new experimental models or extended the studies to new pharmacological properties. 

Among the main biological activities, the anesthetic one is the main reason why *A. oleracea* has been used since ancient times to relieve toothache [90]. To date, the anesthetic mechanism of action is well established, depending on *N*-alkylamide spilanthol [91], which increases the release of gamma-aminobutyric acid (GABA) [92]; interacts with transient receptor potential vanilloid type 1 (TRPV1) and TRPA1 receptors [93]; stimulates the synthesis of prostaglandins; activates the opioidergic [94], serotoninergic, and GABAergic systems [95]; and blocks the voltage-gated Na^+^ channels [11].

The cytotoxic activity of a hexanoic extract, EO, and spilanthol was tested on various cell lines, such as the human fibroblast cell line (NHF-A12) and human tumor cell lines (A375, MDA-MB 231, HeLa, and V79). The data showed moderate cytotoxic activity, with an accentuated action on the cancerous cell lines. The IC_50_ values of the EO were 266.3, 87.80, and 130.9 μg/mL against NHF-A12, MDA-MB 231, and A375, respectively. The IC_50_ values of spilanthol were instead 166.0, 526.2, and 287.2 on the same cell lines [12,96].

Rondanelli et al. [11] have summarized some scientific evidence regarding the anti-inflammatory activities of *A. oleracea*. In detail, it is reported that *A. oleracea* and its main compounds can favor the anti-inflammatory action in different experimental models with various mechanisms. Furthermore, Stein et al. confirmed the anti-inflammatory activity of *A. oleracea* products through in vitro and in vivo models, with a decrease of chymase, nitric oxide (NO), catalase (CAT) and superoxide anion (SOD) activity and chymase expression. Furthermore, they significantly inhibited edema formation, NO production, and cell tissue infiltration in the formalin test, without causing renal and hepatic toxicity [97]. Moreover, jambù EO and spilanthol counteracted inflammation in LPS-activated BV-2 cells. Treatment with EO showed a very strong effect on the two anti-inflammatory enzymes iNOS and COX-2 and reduced the expression of IL-1β and TNF-α. In contrast, spilanthol significantly reduced only the IL-1β expression [12].

Regarding the antioxidant activity, Rahim et al. summarized the tests performed in cells and cell-free systems conducted up to 2021. Most of the reported studies showed high antioxidant activity in all parts of the plant. The results referred to many assays, such as TBARS and SOD, in addition to the DPPH, which is the most frequently reported. It seems that the antioxidant capacity depends on the presence of flavonoids, phenolic compounds, and triterpenoids. Phenolic compounds have demonstrated strong protective effects in SH-SY5Y cells that were exposed to H_2_O_2_ by upregulating SIRT1 and FoxO3a expressions [98].

The antimicrobial activity of *A. oleracea* may be highly species-specific, as it has been tested in vitro against several bacteria and fungi, showing positive results only against some of them, such as *Salmonella typhi* (MIC of 31.25 µg/mL), *Streptococcus mutans*, and *Lactobacillus* species (zones of inhibition ranging between 21 and 29 mm at 20 mg/mL), *Staphylococcus aureus* (MIC of 1.250 µg/mL)*, Bacillus subtilis* (MIC of 1.250 µg/mL), *Escherichia coli* (MIC of 1.250 µg/mL)*, Pseudomonas aeruginosa* (MIC of 1.250 µg/mL), and *Candida albicans* (MIC of 2.50 µg/mL), [99,100,101]. *A. oleracea* also showed antibiofilm potential against pre-formed *S. mutans* biofilms (MIC of 125 µg/mL) [102].

In recent years, the healing activity of *A. oleracea* and its influence in the collagen content and organization have been tested in rats. Yamane et al. showed that jambù helped in collagen deposition and decreased the time of the healing process [61]. Moreover, de Souza Moro et al. demonstrated that *A. oleracea* increased the molecular organization and content of collagen [103].

The use of jambù in folk medicine as an aphrodisiac has led research groups to study its sexually stimulating action [104,105,106]. Lira Batista et al. analyzed the articles published up to 2021 in this regard, and they assumed that jambù works both by topical and oral administration, with a different mechanism of action [107]. Topically the aphrodisiac action probably depends on the tingling sensation on the glans caused by the inhibition of potassium channels and on the release of NO and prostacyclin. However, for the oral effect, it could depend on the increase of testosterone levels, even if the precise mechanism of action is not yet known.

To assess the antiwrinkle activity of the plant, a serum containing an *A. oleracea* extract was tested in human volunteers, and it showed a positive effect on skin hydration levels, improving the facial expression lines and wrinkles on both eye and lip contours [108].

*A. oleracea* also showed anthelmintic activity, as extracts results were effective against both the cestodes *Taenia tetragona* and *Raillietina tetragona* and the nematode *Ascaridia perspicillum* [55]. In a study, an ethanol extract of *A. oleracea* showed a spasmolytic action in an isolated rat ileum. The mechanism of action was correlated to its ability to cause ileal smooth muscle relaxation by blocking voltage-dependent calcium channels [109].

Among the biological activities of *A. oleracea*, there is also its ability to interact with the enzyme tyrosinase. It was found that the inhibition and the activation of the enzyme strongly depends on the amount of spilanthol in the extract tested. The hexane fraction (84% spilanthol) showed activation capacity towards the enzyme, while the dichloromethane fraction showed inhibitory activity [110].

In a screening of several extracts of different parts of twenty-three plant species, it was found that *A. oleracea* also possesses a certain antimalarial activity. In particular, the aqueous extract of its flowers showed moderate activity against *Plasmodium falciparum*, the leading cause of the development of severe and life-threatening malaria [111].

As regards the strong diuretic action of *A. oleracea* and spilanthol, it has long been known that they are able to inhibit the Na^+^/K^+^/Cl^−^ co-transporter in the ascending loop of nephron, thus increasing natriuresis and kaleuresis. The mechanism of action has recently been investigated in depth [112]. It was found that exposure to spilanthol reduces the basal phosphorylation level of NKCC2, whose activity drives the urine-concentrating mechanism, in freshly isolated kidney slices. In addition, it inhibited vasopressin induced AQP2 traslocation in mouse CD cells, suggesting that spilanthol might act also as an ‘acquaretic’. Finally, at the cellular level, spilanthol rapidly reduced or reversed basal and agonist-increased cAMP levels through a mechanism involving increases in intracellular Ca^2+^ [112]. Jambù treatment reduces gastric lesions, inducing cellular proliferation [113], and showed antispasmodic action in gastrointestinal disorders [109]. These effects could be associated with the increase of cellular proliferation, maintenance of gastric mucus, reduction of inflammatory process, and modulation of the antioxidant mechanisms due to the presence of rhamnogalacturonan (RGal) [114].

## 11. Insecticidal Activity

### 11.1. Insect Vectors

*A. oleracea* has shown promising insecticidal activity against a rather wide range of insect species. Among them, mosquitoes represent the main arthropod vectors of human pathogens worldwide [115]. The *A. oleracea* extract has shown to be effective against two malaria vectors, *Anopheles stephensi* (Liston) (Diptera: Culicidae) [116] and *An. culicifacies* (species C) (Giles) (Diptera: Culicidae) [116], the brancroftii filaria vector *Culex quinquefasciatus* (Say) (Diptera: Culicidae) [16,116], and one of the chikungunya fever vectors *Aedes aegypti* (L.) (Diptera: Culicidae) [16,17,113]. The use of *A. oleraceca* appears to be more efficient than other plant extracts, as demonstrated by Pandey et al. [116]. *A. oleracea* hexane extract proved to be the most effective in inducing complete lethality at minimum concentrations [116]. For *An. culicifacies* larvae, the LC_50_ and LC_90_ were 0.87 and 1.92 μg/mL, respectively; for *An. stephensi*, the LC_50_ and LC_90_ required were 4.57 and 7 μg/mL, respectively; for *Cx. quinquefasciatus*, LC_50_ and LC_90_ were 3.11 and 8.89 μg/mL [14,116]. Subsequent studies focused on other extracts, such as *A. oleracea* EO. EO results were more effective at higher doses, as seen by Benelli et al. [117]. LC_50_ was 42.2 μg/mL and LC_90_ was 73.6 μg/mL on *Cx. quinquefasciatus*. A similar result has been reported by Araújo et al. (2018) using hydroethanolic extract (LC_50_ = 32.40 μg/mL; LC_90_ = 68.24 μg/mL) [16].

Different extracts achieved different results, as observed by Araújo et al. [17]. The hexane extract from jambù leaves was more toxic against *Ae. aegypti* larvae (LC_50_= 2.23 μg/mL) than the methanolic and ethanolic extracts. The LC_50_ and LC_90_ recorded on *Ae. aegypti* larvae exposed to a solution containing hexane extract were 5.45 and 16.43 μg/mL, respectively. These concentrations were lower compared with the hydroethanolic extract (LC_50_ of 9.53 μg/mL and an LC_90_ of 28.70 μg/mL) and the methanolic extract (LC_50_ of 28.16 μg/mL and an LC_90_ of 44.40 μg/mL) (Table 3).

### 11.2. Insect Pests

Extract of spilanthol from the flowerheads of *A. oleracea* was found to be active against several insect pests. As has been observed by Kadir et al. on *Periplaneta americana* (L.) (Blattodea: Blattidae), the spilanthol exerts insecticidal activity by penetrating the insect integumental system and interfering with the nervous system [118].

The *A. oleracea* extract topical application has mortality effects on three moths: the Egyptian cotton worm, *Spodoptera littoralis* (Boisduval) (Lepidoptera: Noctuidae) (LD_50_ = 68.1 µg/larva, LD_90_ = 132.1 µg/larva) [14] and the tomato leafminer, *Tuta absoluta* (Meyrick) (LD_50_ = 0.13 μg/mg) without affecting non-target organisms [119] and *Plutella xylostella* (L.) (Lepidoptera: Plutellidae), in which was observed increasing mortality over time (LC_50_ = 1.49, 5.14 and 5.04 g/L for spilanthol, crude methanol, and hexane seed extracts, respectively) [120]. A relevant acute toxicity was also detected testing the EO against adult females of the common housefly, *Musca domestica* (L.) (Diptera: Muscidae) (LD_50_ = 44.3 µg/adult, LD_90_ = 87.5 µg/adult) [14].

Interesting results have also been obtained by treating the feeding substrate, as in the assay against two insect pests, *Myzus persicae* (Sulzer) (Aphididae: Hemiptera), and *Lipaphis erysimi* (Kaltenbach) (Aphididae: Hemiptera). A reduction in insect fecundity was noted [15]. The toxicity of *A. oleracea* extract was noted by Ogban et al. on *Sitophilus zeamais* (Motschulsky) (Coleoptera: Curculionidae) adults, showing significant adult mortality when concentration and days of exposure increased [121]. All these studies demonstrate the possible use of jambú as an insecticidal agent and represent the scientific basis for the industrial exploitation of the EO in the preparation of green insecticides [117].

## 12. Acaricidal Activity

### 12.1. Lethal Effects against Ticks

*Rhipicephalus microplus* (Canestrini) (Ixodida: Ixodidae) is the most economically important cattle ectoparasite in tropical and subtropical regions [122]. Synthetic acaricides have been used indiscriminately to control this parasite over the years, and, over time, resistance has developed in tick populations [123]. Natural plant products are an alternative for the control of ticks. The lethal effect of *A. oleracea* hexane extract was found to be successful against *R. microplus* larvae at concentrations above 1.6 mg/mL (LC_50_ = 0.8 mg/mL) and against engorged females with a LC_50_ of 79.7 mg/mL [124]. High acaricidal activity of the methanolic extract was observed on *R. microplus* and *Dermacentor nitens* (Acari: Ixodidae), with a mortality rate exceeding 90% from concentrations of 1.6 and 6.2 mg/mL, respectively. Those results are in accordance with those obtained by Castro et al., who tested the hexane extract of *A. oleracea* and found an average mortality rate of 93% for *R. microplus* larvae in the treatment with a concentration of 1.6 mg m/L [125]. In these cases, methanol and hexane extracts have the same effect on mortality at similar doses.

Studies have shown the effects of different types of methanol and hexane extracts. The lethal effect of the extract depends on the percentage of spilanthol contained. As tested by Marchesini et al., the dichloromethane fraction with 99% spilanthol showed higher activity, resulting in a larval mortality of *R. microplus* of more than 80% at a concentration of 0.4 mg/mL and reaching 100% at a concentration of 1.6 mg/mL. The methanolic extract of *A. oleracea* was also tested against the immature stages of a Brazilian tick vector *Amblyomma sculptum* (Berlese) (Ixodida: Ixodidae) with 0.187% spilanthol [18]. Test concentrations ranged from 0.4 to 50 mg/mL on larvae and 12.5 to 200.0 mg/mL on nymphs. The methanol extract caused 100% mortality of engorged larvae and nymphs from concentrations of 12.5 and 200.0 mg/mL, respectively. On engorged larvae and nymphs, mortality was 100% from concentrations of 12.5 and 150.0 mg/mL, respectively [126].

### 12.2. Sublethal Effects against Ticks

According to Desneux et al. [5], sublethal effects are upshots (either physiological or behavioral) on individuals that survive exposure to a pesticide (note that the pesticide dose can be lethal or sublethal). In ticks, as a general trend, the acaricide potential of *A. oleracea*-based products was expressed both in terms of lethal and sublethal effects, and it depends on the method of extraction and the content of spilanthol. To the best of our knowledge, sublethal effects have been investigated just on two tick species, the brown dog tick, *Rhipicephalus sanguineus* (Latreille) (Ixodida: Ixodidae) [127], and *Amblyomma cajennense* (Fabricius) (Ixodida: Ixodidae) [128,129]. In *Rhipicephalus sanguineus* (Latreille), significant changes and alterations in the generative and digestive cells of semi-engorged females were recorded when exposed at *A. oleracea* extract doses of 18 mg/mL (LC_50_), 36 mg/mL, and 45 mg/mL, while no effects were reported at lower concentrations. The damage caused by *A. oleracea* extract compromised midgut epithelial cells and female fertility [127]. Regarding *A. cajennense*, studies testing the *A. oleracea* hexane extract showed physiological alterations on both tick sexes. Concentrations of 6.2, 12.5, and 25 mg/mL tested on males showed damage at the reproductive system and alterations of the morpho-histology and physiology of the gland complex, reducing the synthesis of the elements that make up the sperm fluid [127]. Even if these alterations were not enough to affect germ cells, they may hamper the tick reproductive process, reducing their fitness [128]. On females, investigations were done on semi-engorged individuals, and sublethal concentrations were tested at 3.1, 6.2, and 12.5 mg/mL [129]. After the adult immersion test, oocytes and chorion alterations (e.g., loss of the oocyte original shape and a folded and thicker chorion) were recorded in all treatments; in particular, they were more intense at higher concentrations [129]. Furthermore, cytoplasmic disorganization, fewer yolk granules, and the fragmentation of germ vesicle were reported at 3.1 mg/mL, while at 6.2 mg/mL chromatin marginalization in the germ vesicle and large cytoplasmic vacuoles of the oocytes were recorded. At the highest concentration, smaller and ruptured yolk granules were registered [129]. From a histochemical point of view, the period acid-Schiff (PAS) test was used. Females exposed to 3.1 mg/mL of *A. oleracea* hexane extract showed polysaccharides in the oocytes and, in general, moderated alterations compared to the control group. Depending on the oocyte type, different responses were recorded at 6.2 mg/mL, including a loss of yolk granules and irregularities at the plasma membrane. As for the lower concentration, even at 12.5 mg/mL, polysaccharides in oocytes were registered, and again, the responses changed with the oocyte types (e.g., strongly positive yolk granules, no reactions of the germ vesicle and cytoplasm to the PAS, peripherical loss of yolk granules) [129].

### 12.3. Side Effects on Non-Target Species

Our knowledge of the side effects of *A. oleracea* and its main constituents on non-target species is extremely scarce. The zebrafish embryo test is considered a highly sensitive toxicity test of chemical substances. Therefore, it is used to test the toxicity effect of spilanthol on animals. From the study conducted by Ponpornpisit et al., the extract was not toxic on zebrafish [130], but, as observed by de Souza et al., there was a significant increase in egg deposition, which involves fewer resources and more mortality in eggs [131].

## 13. Conclusions

The actual world is dominated by an increasing concern for the use of pesticides. In fact, their excessive usage has led to negative consequences in terms of resistance emergence, human health, and environmental impact. In this regard, botanical pesticides represent promising alternatives to the usually employed insecticides, and *A. oleracea* is a Brazilian plant worthy of considerable attention. It is widely used in traditional medicine to treat several pathologies and has been the subject of several studies due to its numerous biological activities. Among them, the insecticidal property emerged undoubtedly as one of the most promising. From a safety perspective, its use in foodstuffs should reduce the risk to human and animal health from its use as a biopesticide and facilitate the registration process. This review aims at summarizing for the first time data available on *A. oleracea*’s insecticidal and acaricidal properties and to highlight its potential as an innovative source of botanical pesticides, with a focus on the role of spilanthol, the main alkylamide found in this plant. In fact, it emerged that the EO and extracts of jambù are active against different insect vectors, such as *An.* *culifacies, Cx. quinquefasciatus*, and *M. domestica*, which are responsible for the transmission of numerous diseases. Moreover, these *A. oleracea*-derived products also showed acaricidal potential, being effective on tick species such as *A. cajennense* and *R. sanguineus*. These activities are linked to spilanthol, the principal compound involved in the insecticidal and acaricidal action, due to its ability to easily penetrate the insect and mite through the cuticle and affect the central nervous system, disrupting the metamorphosis process. Furthermore, jambù is already widely cultivated for nutraceutical purposes, and for that reason, there is a disposability of plant biomass to be employed on an industrial level to develop botanical insecticide formulations. Since jambù is already widely cultivated in many countries around the world, the cultivated area of this crop could be easily extended for agrochemical purposes. Even if this work underlines the potential of *A. oleracea* as a new and effective source of insecticidal and acaricidal agents, it also points out that it is still necessary to investigate not only the non-target effects of *A. oleracea*-derived products but also the development and study of encapsulating systems able to convey these products.

## Figures and Tables

**Table 1:** Studies on the cultivation techniques of *Acmella oleracea*.

Cultivation Technique	Soil Treatment/Medium	Shoot Formation	Plant Productivity	Minerals/ Secondary Metabolites	References
In field	mineral fertilizer	nr ^a^	4.40 kg m^−2^	high N and K content	[37]
In field	organic fertilizer	nr	2.78 kg m^−2^	high N and P content	[37]
In field	organic fertilizer and vermicompost	nr	2.90–3.87 kg m^−2^	nr	[38]
In field	organic fertilization	nr	nr	high levels of total phenolics and carotenoids	[40]
In field	conventional fertilizer	nr	nr	high levels of nitrates, vitamin C and organic N	[40]
In field	biostimulant	nr	enhanced of 22–25%	maintained levels of alkylamides and polyphenols	[41]
Hydroponic culture	nitrogen-rich nutrient solution	nr	enhanced (at 21 mmol L^−1^ of N)	nr	[42]
In vitro (axillary buds)	MS ^b^ medium supplemented with 2.0 mg L^−1^ of *N*^6^-benzyladenine	multiple-shoot formation from each axillary bud	nr	nr	[43]
In vitro (nodal segments)	MS medium supplemented with 0.1 mg L^−1^ kinetin	93.33% rooted plants	nr	nr	[44]
In vitro (nodal segments)	MS medium with 5.0 mg dm^−3^ of BAP ^c^	97% of shoot regeneration	nr	nr	[45]
		multiple-shoot regeneration	nr	nr	[46]
In vitro (nodal segments)	MS medium without growth regulators	nr	nr	spilanthol content: 0.98%	[48]
Acclimatized	in soil	nr	nr	spilanthol concentration: 9.54%	[48]
In field	organic fertilization	nr	nr	spilanthol concentration: 4.65%	[48]
In vitro (nodal segments)	MS medium with the use of 0.125 mg L^−1^ of the BAP	average of 2.2 sprouts/explant	nr	nr	[49]

^a^ nr, not reported; ^b^ Murashige and Skoog; ^c^ 6-benzylaminopurine.

**Table 2:** Secondary metabolites detected in *Acmella oleracea*.

Compound		Reference
Triterpenoids	3-acetylaleuritolic acid	[64]
	*β*-sitostenone	
	stigmasterol	
Steroidal glycosides	stigmasteryl-3-*O*-*β*-D-glucopyranosides	[64]
	β-sitosteryl-3-*O*-*β*-D-glucopyranoside	
Phenolics	vanillic acid	[64]
	*trans*-ferulic acid	
	*trans*-isoferulic acid	
Fatty acids	*n*-hexadecanoic acid	[46]
	*n*-tetradecanoic acid	
Coumarins	scopoletin	[64]
Volatile compounds	*β*-pinene, myrcene, (*E*)-caryophyllene, caryophyllene oxide, germacrene D, *β*-phellandrene, spilanthol and acmellonate	[65]
		[14]
		[12]
*N*-alkylamides	isobutylamides	[67]
	methylbutylamides phenylbutylamides	[68]

**Table 3:** Insecticidal activity of *Acmella oleracea*-borne products on insect pests and vectors. EO: essential oil.

**Target**			
**Species and Instar**	Treatment	Efficacy	References
*Periplaneta americana* adults	*A. oleracea* Spilanthol crude extract	LC_50_ =2.46 μg/g	[115]
*Anopheles culifacies* late third/early fourth instar larvae	*A. oleracea* hexane extract	LC_90_ = 1.92 μg/mL LC_50_ = 0.87 μg/mL	[113]
*Anopheles stephensi* late third/early fourth instar larvae	*A. oleracea* hexane extract	LC_90_ = 7 μg/mL LC_50_ = 4.57 μg/mL	[113]
*Culex quinquefasciatus* late third/early fourth instar larvae	*A. oleracea* hexane extract	LC_90_ = 8.89 μg/mL LC_50_ = 3.11 μg/mL	[113]
*Tuta absoluta* second instar larvae	*A. oleracea* hexane extract	LC_80_ = 2.94 μg/mL LC_50_ = 1.83 μg/mL	[116]
*Pluella xylostella* second instar larvae	*A. oleracea* crude extract, methanol and hexane extract	LC_50_ = 1.49 × 10^−3^ μg/mL LC_50_ = 5.14 × 10^−3^ μg/mL	[117]
Sitophilus zeamais adults	*A. oleracea* plant powder	100% mortality after 96 h of exposure to 1% (w/w)	[118]
Culex quinquefasciatus third instar larvae	*A. oleracea* hydroethanolic extract	LC_90_ = 1.92 μg/mL LC_50_ = 32.40 μg/mL	[13]
*Aedes aegypti* third instar larvae	*A. oleracea* hexane extract, topical application	LT_90_ = 57.05 h LC_50_ = 44.3 µg/adult	[13]
Culex quinquefasciatus third instar larvae	A. oleracea EO	LC_90_ = 68.24 μg/mL LC_50_ = 42.2 × 10^−4^ μg/mL	[11]
Musca domestica adult females	*A. oleracea* EO topical application	LC_90_ = 73.6 × 10^−4^ mg/mL LC_50_ = 44.3 µg/adult	[11]
Spodoptera littoralis third instar larvae	*A. oleracea* EO topical application	LC_90_ = 87.5 µg/adult LC_50_ = 68.1 µg/larva	[11]
Myzus persicae nymphs	*A. oleracea* ethanol extract on kale leaves: 0.01 g/mL	LC_90_ = 132.1 µg/larva LT_50_ = 3.29 h	[12]
Liphapis erysimi nymphs	*A. oleracea* ethanol extract on kale leaves: 0.01 g/mL	LT_90_ = 24.85 h LT_50_ = 8.85 h	[12]
*Aedes aegypti* third instar larvae	*A. oleracea* hydroethanolic extract (1), hexane extract (2), methanolic extract (3)	LC_90_ = 87.5 µg/adult LC_50_ = (1) 28.42 μg/Ml (2) 23.2 μg/Ml (3) 39.67 μg/mL LC_90_ = (1) 63.45 μg/mL (2) 39.98 μg/mL (3) ld>(3) 63.34 μg/mL	[14]

## Data Availability

Not applicable.
